# Supplementary material for: Population Dynamics of Drosophila suzukii in Coastal and Mainland Sweet Cherry Orchards of Greece
Source: Insects. 2020 Sep 10;11(9):621. doi: 10.3390/insects11090621 (PMC7564280; doi:10.3390/insects11090621)
Supplement: Supplementary file 1 [file insects-11-00621-s001.pdf]

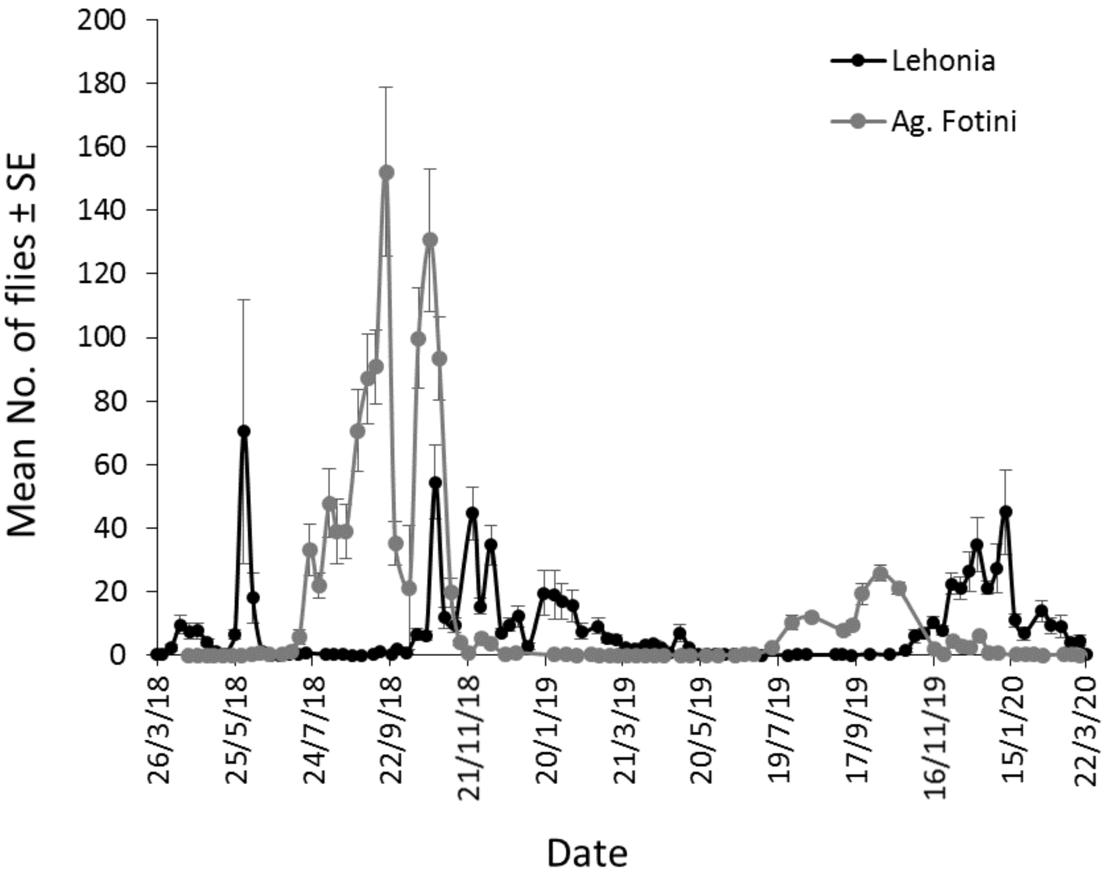

Figure S1. Mean number of total flies per trap in Lehonia and Agia Fotini for each observation date from 26 March 2018 to 22 March 2020.

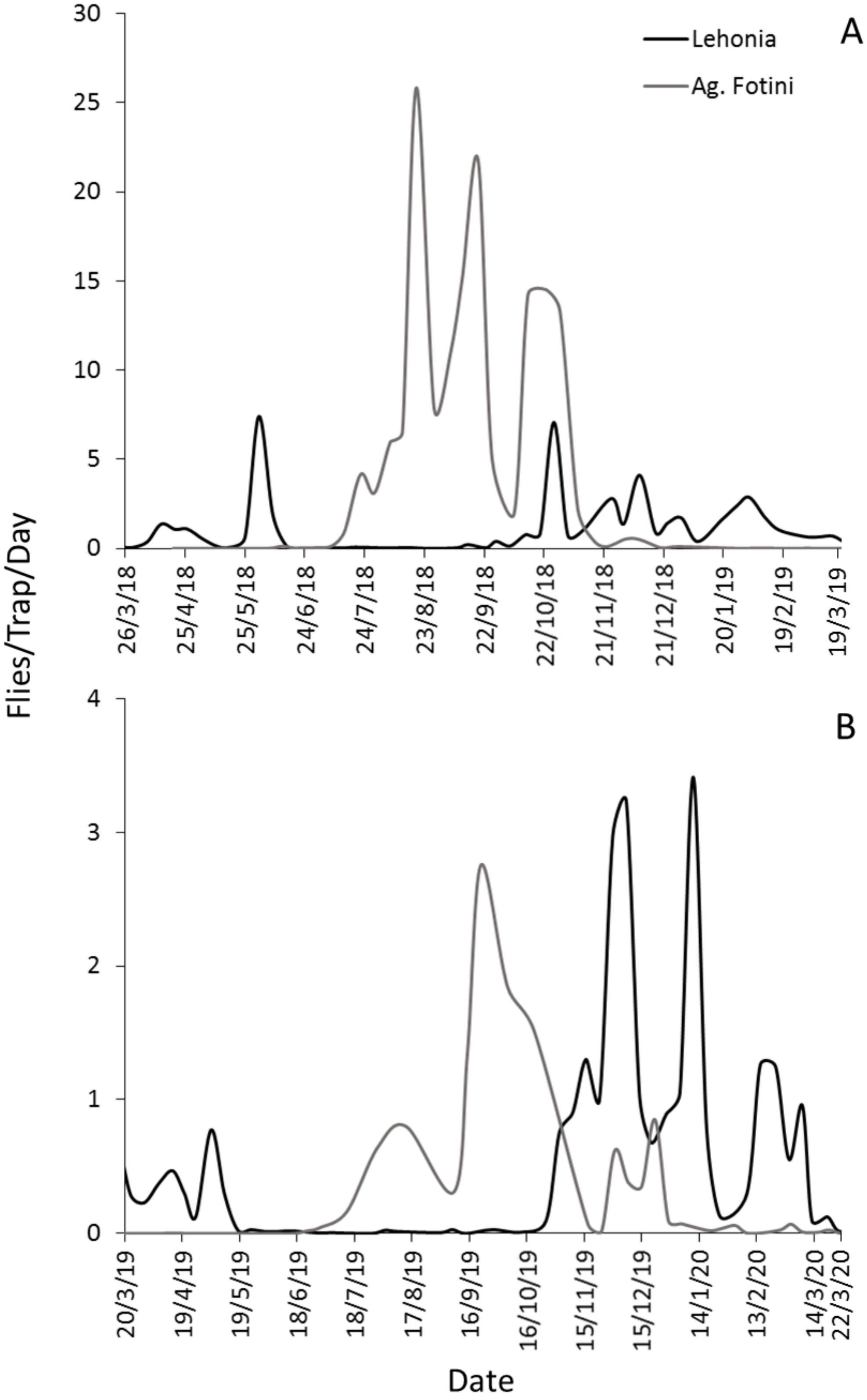

Figure S2. Adult captures per trap per day in the two geographic locations Lehonia and Agia Fotini from 26 March 2018 to 19 March 2019 (**A**) and from 20/3/2019 to 22 March 2020 (**B**).

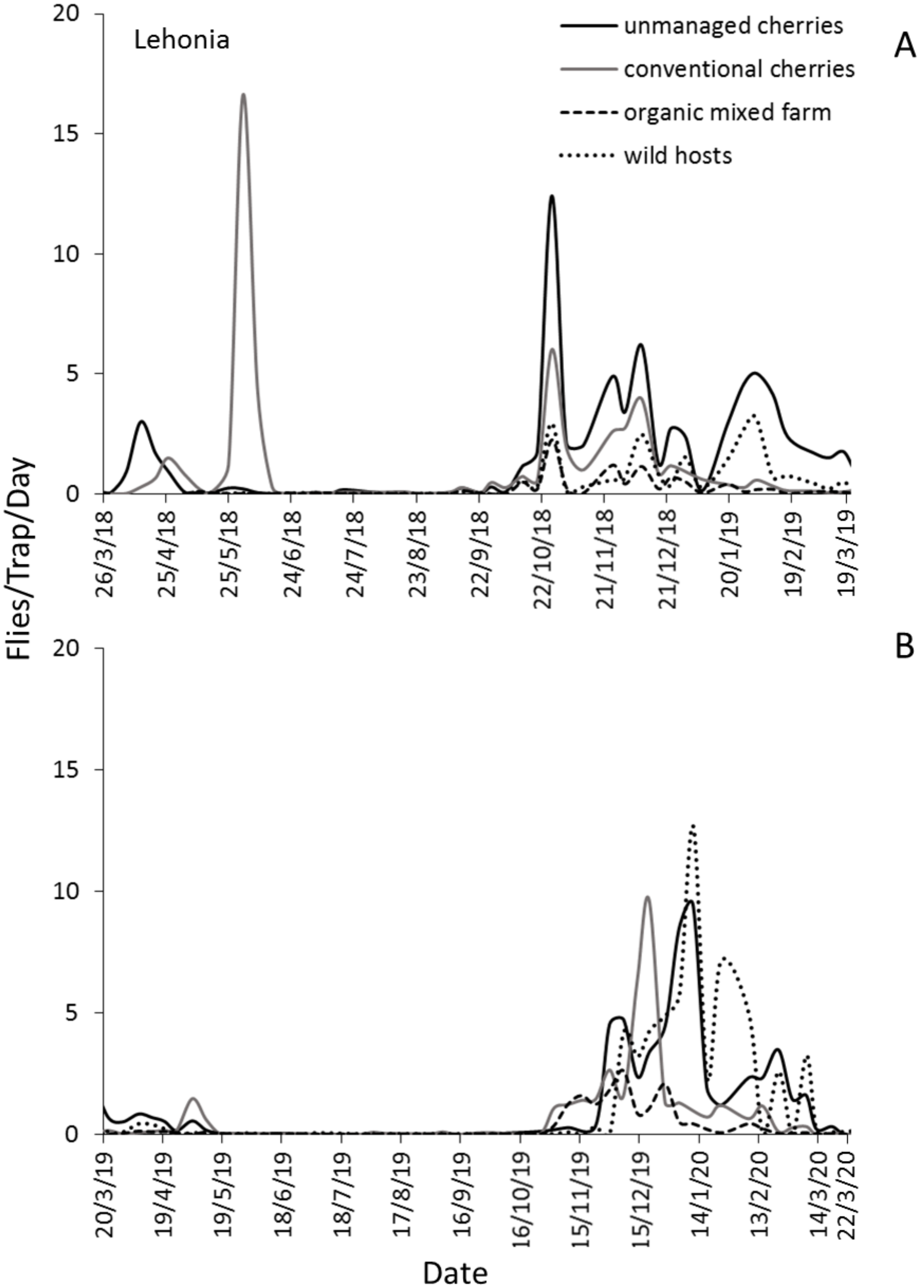

Figure S3. Adult captures per trap per day for unmanaged and conventional cherry trees, an organic mixed orchard and wild vegetation in Lehonia from 26 March 2018 to 19 March 2019 (**A**) and from 20 March 2019 to 22 March 2020 (**B**).

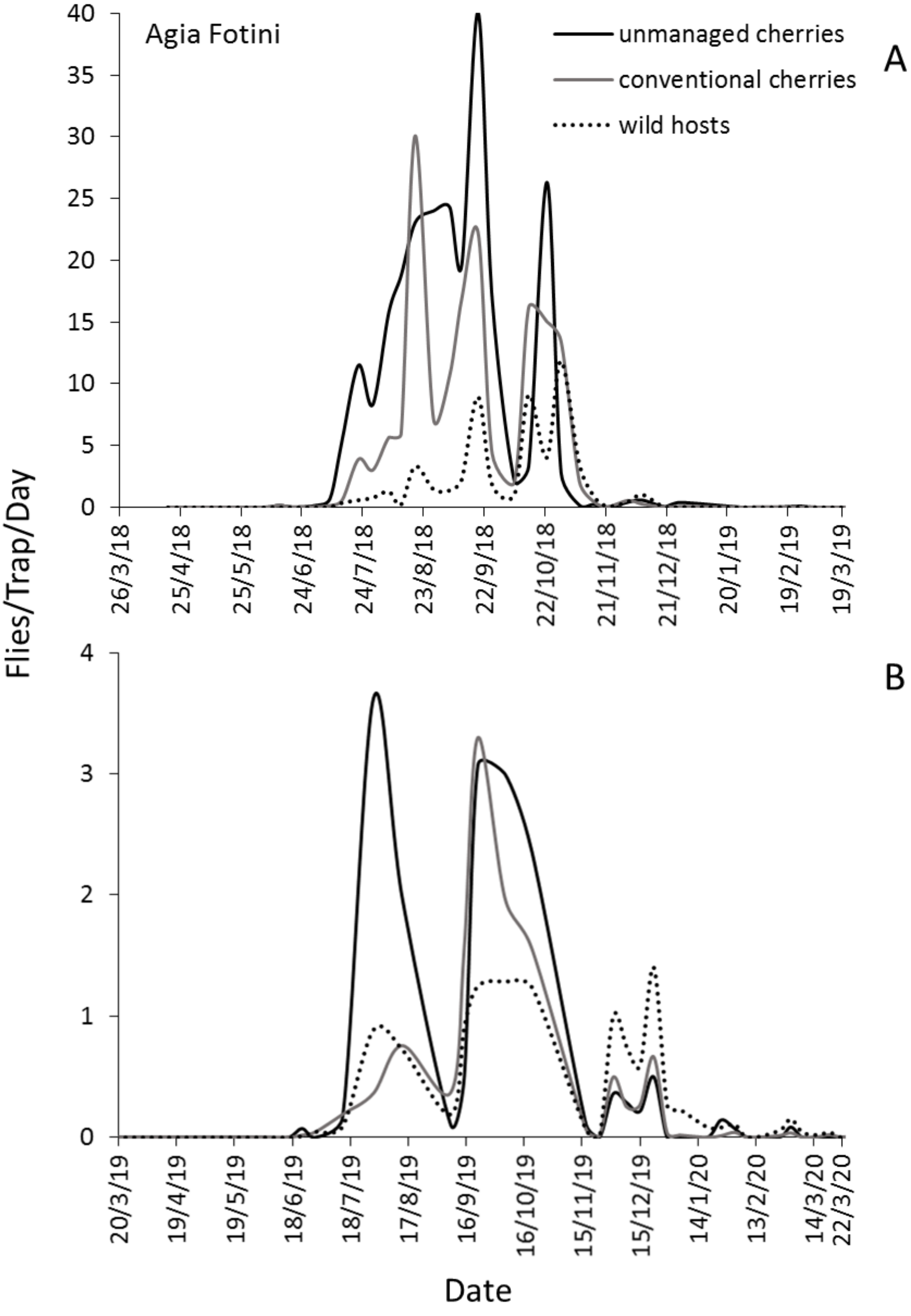

Figure S4: Adult captures per trap per day for unmanaged and conventional cherry trees, and wild vegetation in Agia Fotini from 26 March 2018 to 19 March 2019 (**A**) and from 20 March 2019 to 22 March 2020 (**B**).
